# Review of novel human β‐coronavirus (2019‐nCoV or SARS‐CoV‐2) from the food industry perspective—Appropriate approaches to food production technology

**DOI:** 10.1002/fsn3.1892

**Published:** 2020-09-18

**Authors:** Mohammad Goli

**Affiliations:** ^1^ Department of food science and technology Isfahan (Khorasgan) Branch Islamic Azad University Isfahan Iran; ^2^ Laser and Biophotonics in Biotechnologies Research center Isfahan (Khorasgan) Branch Islamic Azad University Isfahan Iran

**Keywords:** enriching, fenton reaction, good manufacturing practices, human β‐coronavirus, industrial food fortifying, survival conditions disruption

## Abstract

Coronaviruses, enveloped nonsegmented positive‐sense RNA viruses, can affect the respiratory and digestive systems of humans and a variety of birds and mammals. The primary target cells of coronaviruses compromise the respiratory and gastrointestinal region epithelial cells due to their cell features and delivery through fomites, airborne, or fecal–oral routes. Some functional food sources due to having crucial chemical compounds may help individuals to overcome this infection by modulating the body's immune system, generating antiviral activity against the infection, and reducing other respiratory problems. The purpose of this study was to review these coronaviruses, especially SARS (because of its very similar gene sequence to the 2019‐nCoV or SARS‐CoV‐2), from the perspective of appropriate approaches to food production technology, including following good food safety practices in food production lines; avoidance of underheating in the processing of swine and the other meat products; uncertainty about the safety of frozen or refrigerated meat products; providing unfavorable environmental conditions for coronavirus survival (minimum heat treatment, e.g., low‐temperature long time and greater for liquid food products, pH ≤ 3, minimum storage relative humidity); production of industrial foods fortified and enriched with vitamin D, C, B3, K, amino acid L‐tryptophan, nicotinamide adenine dinucleotide (NAD^+^), and tannins; and preventing the production of industrial foods fortified or enriched with mineral supplements that participate in the Fenton reaction in the human body. Considering these aspects during times and places of coronavirus, prevalence will be essential for preventing further outbreaks at the community level.

## Graphical abstract

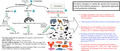

## 1 INTRODUCTION

Coronaviruses are surrounded nonsegmented positive‐sense RNA viruses relating to the family Coronaviridae, the subfamily Orthocoronavirinae [divided into 4 genera, i.e., α, β, δ, and γ‐coronavirus] that extendedly spread in mammals and birds (ICTV, 2020). The primary target cells of coronaviruses compromise the respiratory and gastrointestinal region epithelial cells due to their cell features and delivery through fomites, airborne, or fecal–oral routes (Yin & Wunderink, 2018). The current 2019‐nCoV, which causes an acute respiratory disease case, is closely related to SARS‐CoV, that is, within the genus β‐coronavirus (WHO, 2020b). Since there is little epidemiological and pathogenic information about this virus, a genetic analysis of this virus is very similar to SARS‐CoV and is sometimes referred to as SARS‐CoV‐2 (ECDC, 2020). Therefore, animal and human origin safety authorities in the EU/EEA countries ought to follow the recommendations used for SARS‐CoV and MERS‐CoV outbreaks (ECDC, 2020). Clinical manifestations of β‐coronaviruses including fever, nonproductive cough, nasal congestion, and fatigue start after less than a week of infection. Lymphopenia and elevation of inflammatory markers including C‐reactive protein and proinflammatory cytokines considered as diagnostic clinical laboratory manifestation so that anthogenesis of 2019‐nCoV is genetically similar to SARS‐CoV‐1 and MERS‐CoV, 79% and 50%, respectively (Kouhpayeh et al., 2020; Lu et al., 2020). Although most of the human coronavirus infections are mild and asymptomatic, the pandemics of the two β‐coronaviruses, that is, SARS‐CoV (β‐coronavirus, subgenus Sarbecovirus, 2002–2003, case fatality rate 10%) (Drosten et al., 2003; Ksiazek et al., 2003; Kuiken, Fouchier, & Schutten, 2003) and MERS‐CoV (Betacoronavirus, subgenus Merbecovirus, 2012, case fatality rate 35%) (De Groot et al., 2013; Zaki, van Boheemen, Bestebroer, Osterhaus, & Fouchier, 2012) have caused vast fatal human pneumonia, especially in the immunocompromised people, the cardiopulmonary patients, and the old ones and adolescents (ECDC, 2020; WHO, 2002b). SARS‐CoV‐infected humans due to eating animals infected with bat coronavirus, that is, Himalayan palm civets, Chinese ferret badgers and raccoon dogs sold for food, can cause animal‐to‐human and human‐to‐human transmission. The most relevant animal reservoirs of human MERS‐CoV are dromedary camels that caused human–human infections, especially in healthcare environments, in Saudi Arabia, 2012. (Hui et al., 2018; Park, Jung, & Kim, 2018).

Some functional food sources due to having crucial chemical compounds, for example, onion (quercetin, thiosulfinates, and anthocyanins), garlic (diallyl disulfide, alliin, polyphenols, and proteins (QR‐1, QR‐2, and QR‐3)), barberry (berbamine and berberine), tea plant (Catechins, quercetin, gallic acid, and theaflavin‐3,3’‐digallate), papaya (caricaxanthin, violaxanthin, zeaxanthin, carpaine, dehydrocarpaine I and II, and cardenolide), bitter orange (polysaccharides and polyphenolic compounds), turmeric (curcumin), fruit and leaves (terpenoids, anthocyanins, and steroids), seeds (isoflavones, flavonoids, phytosterols, organic acid, and saponins), liquorice (glycyrrhizin) wolfberry (polysaccharide–protein complexes, and phenolic compounds), mango (flavonoids, xanthones, i.e., mangiferin, phenolic acids, and triterpenes), mulberry (carotene, vitamin B1, folic acid, folinic acid, vitamin D, polyhydroxylated alkaloids, glycoprotein, anthocyanins, benzofurans, and stilbenes), black cumin (quinones, alkaloids, and saponins), long and black pepper (piperine), plum (anthocyanins and protocatechuic acid), guava (phenolic, flavonoid, carotenoid, terpenoid, and triterpenes), pomegranate (anthocyanins, fatty acids, alkaloids, and vitamins), and ginger (essential oil, crude fiber, proteins, fatty oils, and carbohydrates) may help individuals to overcome this infection by modulating the body's immune system, generating antiviral activity against the infection, and reducing other respiratory problems. Immunomodulators not only enhance humoral and cell‐mediated immunity but also activate nonspecific immune responses such as activation of the natural killer (NK) cells, macrophages, granulocytes, and complement systems, which enhances resistance to infections nonspecifically. Activation of these important immune cells results in the production of various molecules such as interferons, cytokines, and chemokines involved in the enhancement of immune responses. In most SARS autopsies, the numbers of several immune cells present in the spleen, including lymphocytes (CD^4+^, CD^8+^, CD^20+^), dendritic cells (DT), and NK cells, decreased as well as the size of macrophages increased by more than 100%, and macrophages and T lymphocytes have been reported to be infected with high viral loads (Fan et al., 2020).

This review aimed to summarize for the first time all available data related to emerging novel SARS‐CoV‐2 (i.e., 2019‐nCoV) and SARS‐CoV (because of its very similar clinical presentation and highly comparable gene sequence to 2019‐nCoV or SARS‐CoV‐2), and MERS‐CoV from the perspective of the appropriate approaches to food production technology in three parts including, avoiding consumption of any type of suspicious raw materials with the potential to transmit coronavirus in food formulation (due to livestock and poultry are potential carriers of SARS‐CoV and animal‐to‐human and human‐to‐human transmission is possible), disrupting the survival conditions of the coronavirus, and enriching or fortifying the industrial food formulations with effective components in improving COVID‐19. Considering the appropriate approaches to food production technology, which will be effective in preventing further outbreaks at the community level.

## 2 CORONAVIRUS FROM THE PERSPECTIVE OF APPROPRIATE APPROACHES TO FOOD PRODUCTION TECHNOLOGY

### 2.1 Possible origin and target organisms of the 2019‐nCoV

SARS and MERS emerged in 2003 and 2012, affected thousands of human lives, while SADS damaged the swine industry in 2017. These viruses have well‐known features and are all notably human–livestock pathogens originated from bats introduced in China. Thus, it is extremely likely that future SARS‐ or MERS‐like coronavirus prevalence will originate from bats, and there is an increased possibility that this will happen in China. For minimizing the impact of future outbreaks in the world, the study of bat coronaviruses is important for the detection of early warning signs (Yi, Kai, Zheng‐Li, & Peng, 2019). On 31 December 2019, a cluster of pneumonia cases of unknown etiology, jointed to Seafood Wholesale Market (Contains fish and live different animal species) was reported by Wuhan municipal health commission in Wuhan in China. On 9 January 2020, the China CDC detected 2019‐nCoV as the causative agent, with genome sequence was well known. The newly identified virus sequence analysis has highly resembled SARS‐CoV, and even it is also mentioned as SARS‐CoV‐2 (ECDC, 2020). Therefore, in these critical cases, some preventive measures are strongly recommended (Table 1).

**Table 1:** Preventive strategies to combat the spread of β‐coronavirus from the perspective of avoiding consumption of any type of suspicious raw materials with the potential to transmit coronavirus in food formulation

Preventive measures	Scientific reasons	References
Avoiding consumption or having direct contact with animal origin raw or inadequately cooked food products and following good food safety practices in food production lines	China's market of live animals, including fish and other animal species, reported on 31 December 2019	(EDCD, 2020)
	The emergence of the 2019‐nCoV has recently added to the list of problematic emerging pathogens in the 21st century, which was suspected to originate from the persons exposed to a seafood or wet market in Wuhan, China, suggesting animal‐to‐human transmission	(WHO, 2020b; Huang et al., 2020)
	SARS, MERS, and SADS are all highly pathogenic to humans and livestock	(Yi et al., 2019)
Avoid producing, importing, and eating any abnormal meat products derived from bat, mouse, dog, and cat meat that could be potential sources of β‐coronavirus transmission	All coronaviruses are classified into four genera such as α‐coronavirus and β‐coronavirus, both of which infect mammals, γ‐coronavirus that infects avian species, and δ‐coronavirus that infects both mammalian and avian species. They are a large enveloped virus with a positive‐sense single‐stranded RNA genome of about 26 to 33 kb that is distributed broadly among birds, humans, and other mammals such as camels, bats, mice, dogs, and cats	(Lu et al., 2020)
	The β‐coronaviruses including SARS‐CoV and MERS‐CoV are believed to have originated from bats and have subsequently been transmitted to mammals, that is, humans and livestock	(Cui, Li, & Shi, 2019; Daniell, Rai, & Xiao, 2019)
	Safety authorities in the EU/EEA countries will follow the recommendations used for SARS‐CoV and MERS‐CoV because the 2019‐nCoV infection caused clusters of severe respiratory illness greatly resembling SARS‐CoV, with high mortality	(ECDC, 2020; Huang et al., 2020)
Nonconsumption of any animal by‐product powder (such as blood powder as a coloring agent, eggshell, gelatin, or keratin) with transient potential to harbor coronaviruses, especially β‐coronavirus in human, livestock, poultry, and aquatic food formulations	Potential for transmission of SARS‐CoV, MERS‐CoV, and SARS‐CoV−2 (2019‐nCoV) through blood products has been seen repeatedly. Viral RNA was detected in the plasma or serum from COVID−19 patients	(Chang et al., 2020)

## 3 ENVIRONMENTAL CONDITIONS (HEAT TREATMENT, pH, PROTEIN PROTECTIVE EFFECT, AND RELATIVE HUMIDITY) EFFECTS ON 2019‐NCOV STABILITY AND SURVIVAL

Coronaviruses are vulnerable to acid pH, basic pH, and heat (Rabenau et al., 2005) but seem to be more stable at 4℃ (Lamarre & Talbot, 1989). The infectious titer of the virus did not display any significant reduction after 25‐cycle thawing and freezing (Lamarre & Talbot, 1989). Usually, treatment with 60℃ for 15–30 min is sufficient for the reduction of SARS‐CoV in plasma without cells, and inactivation could be achieved by treatment with 60℃ for 10 hr for plasma products. In another study, heating at 56℃ for 25 min reduced the MERS‐CoV by more than 4 log_10_ due to protein heat denaturation in blood products, it could only be used in manufacturing blood products derived plasma (Chang, Yan, & Wang, 2020). The low‐temperature long time (LTLT) heat treatment, that is, 60℃ for 30 min in liquid foods such as milk, caused no infectious virus remaining, regardless of the presence of the protein additive. While 56℃ for over 30 min in absent of protein caused the virus titer reached below the detection limit with a reduction factor > 5.01 log_10_ and in the presence of protein, for example, fetal calf serum 20%, the reduction factor was only 1.93 log_10_. At the refrigerated conditions, that is, 4 ℃ (control), there was no loss of infectious titer; namely, the reduction factor was zero (Rabenau et al., 2005). The SARS‐CoV is more thermal‐ and chemical‐sensitive and has significantly greater environmental stability compared to the human CoV‐229E. In a dried environment, SARS‐CoV retained residual infectivity even after 6 days, while human CoV‐229E completely lost its infectivity within 24 hr (Rabenau et al., 2005). On a variety of substances, HCoV‐229E can survive from 2 hr to 9 days. A higher temperature, that is, 30℃ or 40℃, persistence duration time of highly pathogenic, that is, MERS‐CoV, transmissible gastroenteritis virus (TGEV) and murine hepatitis virus (MHV), was seriously reduced (Kampf, Todt, Pfaender, & Steinmann, 2020). The stability of human CoV‐229E infectivity was at a maximum state at pH 6.0 regardless, different incubation temperatures either 4 or 33℃. However, the influence of pH was more noticeable at higher temperatures. Viral infectivity was entirely lost after a 14‐day incubation time at higher than 22℃ but remained relatively constant at 4°C for the same length of time. Besides, 25‐cycle and 15‐cycle thawing–freezing (at least 2 hr at −70℃ and then thawed in a 37℃ water bath) did not have any reduction in human CoV‐229E and MHV‐A59 infectivity, respectively. The pH and temperature are two important and easily controllable factors for other viral growth factors. Coronaviruses' infectivity had decreased as they exposed to acidic pH values at 37℃ but had been relatively stable at 4℃ (Lamarre & Talbot, 1989). The optimized conditions, for example, appropriate temperature and pH value, can facilitate the coronaviruses molecular properties (Lamarre & Talbot, 1989). The optimal stability of viral infectivity was observed at pH 6.0 at both 4 and 33℃, while in incubation temperature 4℃ survival of viruses was more at pH values > 6.0. Indeed, viral infectivity was undetectable after exposure to pH 4.0 or 9.0 at 33℃, whereas at 4℃ incubation temperature, 93 and 84% of viral infectivity remained after exposure to these pH values 4.0 or 9.0 and buffered pH value 10, respectively. Finally, the best viral activity is in acidic and refrigeratory conditions between pH 5.0 and 8.0 and 4℃, respectively. For example, the avian infectious bronchitis virus is more stable at acidic pH values. The MHV‐A59 was stable for 3 months at 4℃, whereas infectivity was undetectable after 14 days at 22 and 37℃ (Lamarre & Talbot, 1989). Even, at 4℃, the persistence of TGEV and MHV can be raised to 28 days. Notably, SARS‐CoV infectivity was longer even with a higher inoculate. Also, it was shown that at 20–22°C, HCoV‐229E is more persistent at 50% compared to 30% relative humidity (Kampf et al., 2020; Warnes, Little, & Keevil, 2015). Therefore, in these critical cases, some preventive measures are strongly recommended (Table 2).

**Table 2:** Preventive strategies to combat the spread of β‐coronavirus from the perspective of disrupting the survival conditions of the coronavirus

Preventive measures	Scientific reasons	References
Avoidance of inadequate heat treatment (underheating) in the production and processing of swine meat products.	One of the zoonotic SARS‐, MERS‐, or 2019‐nCoV‐like coronaviruses caused swine acute diarrhea syndrome (SADS), which struck the swine industry in 2017	(Yi et al., 2019)
	SARS‐CoV and SADS‐CoV were transmitted from bats to humans or swine.	(Yi et al., 2019)
Uncertainty about the safety of frozen or refrigerated meat products	Freezing and refrigeration procedures (4–10°C) have no loss effects on coronavirus infectious titer The coronavirus seems to be more stable at 4°C The infectious titer of the virus did not show any significant reduction after 25 cycles of thawing and freezing	(Chang et al., 2020; Lamarre & Talbot, 1989)
Apply minimal heat treatments, that is, low‐temperature long time (LTLT) and more for liquid food products	Heat treatments (60℃ for at least 30 min) in protein‐containing food solutions and a minimum temperature of 56℃ in protein‐free food solutions cause denaturing of the secondary structures of proteins and cause an acceptable reduction in MERS‐CoV by 4 log_10_	(Chang et al., 2020; Leclercq, Batejat, Burguiere, & Manuguerra 2014; Rabenau et al., 2005)
Produce food products with pH ≤ 3 (i.e., fermented sausage and fermented dairy products), store at nonrefrigeration temperatures, and preferably consume after a 14‐day shelf life.	The stability of human coronavirus 229E infectivity was at a maximum at pH 6.0 when incubated at either 4 or 33°C Viral infectivity was completely lost after a 14‐day incubation period at 22, 33, or 37°C but remained relatively constant at 4°C for the same length of time Determining the optimum virus growth and storage conditions will facilitate the molecular characterization of this important pathogen	(Lamarre et al., 1989)
Establish preferably low relative humidity and high temperature in food production lines and warehouses.	A higher temperature such as 30°C or 40°C reduced the duration of persistence of highly pathogenic MERS‐CoV, TGEV, and MHV At 4°C, the persistence of SARS‐CoV (more than 28 d)> TGEV and MHV (2 hr–9 d) At room temperature, HCoV‐229E persists more at 50% compared to 30% relative humidity	(Kampf et al., 2020; Casanova, Jeon, Rutala, Weber, & Sobsey, 2010)

## 4 VITAMIN D AND STRENGTHENING THE IMMUNE SYSTEM AGAINST β‐CORONAVIRUS

Vitamin D showed significant effects through binding to the ACE II receptor which mediates acute lung injury in host cells during 2019‐nCoV infection. The high expression of ACE II receptor on the surface of human alveolar epithelial cells significantly facilitates coronavirus internalization and infection (Zhang, Penninger, Li, Zhong, & Slutsky, 2020). Thus, vitamin D deficiency may directly promote hypertension through impacts on members of renin–angiotensin (RAS). Another protective function, which has been proposed for vitamin D, is immunomodulation through suppressing proinflammatory cytokines. Hence, vitamin D supplements may ameliorate the cytokine storm following 2019‐nCoV infection (Al‐Hadad, Neama, & Al‐Mousawi, 2019; Kouhpayeh et al., 2020). In vitamin D deficiency, the ACE II receptor binding sites are exposed on the cell surface which could further enhances the viral entry into the host cells. Thereby, it seems vitamin D is considered as a critical player in capability of 2019‐nCoV infection. And, vitamin D deficiency may affect the immune system because vitamin D enhances the inherent immune system by upregulating expression and secretion of antimicrobial peptides supporting mucosal defense. Also, the protective effect of vitamin D supplementation against respiratory tract infections has been detailed by novel meta‐analyses. Vitamin D cooperates with cells responsible for fighting infection so that insufficient vitamin D could increase the risk of being infected with the coronavirus (Aponte & Palacios, 2017). Therefore, food industry officials and authorities should take immediate action to produce and distribute industrial foods fortified and enriched with vitamin D (Table 3).

**Table 3:** Preventive strategies to combat the spread of β‐coronavirus from the perspective of enriching the industrial food formulations with effective components in improving COVID‐19

Preventive measures	Scientific reasons	References
Immediate production and distribution of industrial foods fortified and enriched with vitamin D	Vitamin D upregulates the expression and secretion of mucosal enhancer antimicrobial peptides, has protecting effects on respiratory tract infections, and directly interacts with cells responsible for fighting deadly coronavirus infection	(Aponte & Palacios, 2017)
Immediate production and distribution of industrial foods fortified and enriched with vitamin C	Vitamin C affects the immune system health, that is, the function of phagocytes, the transformation of T lymphocytes, and the production of interferon In particular, it increased the resistance of chick embryo tracheal organ cultures to infection caused by an avian coronavirus Protecting broiler chicks against an avian coronavirus. Viral respiratory infections in humans are affected by vitamin C levels There is also evidence indicating that vitamin C affects pneumonia Vitamin C is also important for iron absorption, and iron deficiency can increase vulnerability to infections, in general	(Hemila, 2003)
Food fortification with NAD^+^, vitamin B3 (niacin) and L‐tryptophan	2019‐nCoV, almost all procedures lead to or originate from NAD^+^ depletion which cause to decreasing sirtuin 1(SIRT1) SIRT1 deacetylates nuclear proteins using NAD^+^ to regulate the expression of genes including tumor suppressors, cytokines, and proto‐oncogenes and ultimately modulate inflammation, cell survival, and apoptosis mechanisms In 2019‐nCoV patients, the resources of tryptophan—as the raw material for serotonin and NAD synthesis—spend, and in acute respiratory distress syndrome (ARDS), course of the disease serotonin is decreased and hypo‐aldosteronism causes hyponatremia and hypovolemia	(Kouhpayeh et al., 2020; Kume et al., 2007; Matsuoka et al., 1985)
Use vitamin k supplements or vitamin K‐rich food sources in food formulations especially for COVID−19 patients	COVID‐19 may also progress beyond the lungs Vitamin K status is reduced in patients with severe COVID‐19, and low vitamin K status seems to be associated with accelerated elastin degradation and vitamin K administration improves outcome in patients with COVID‐19	(Dofferhoff et al., 2020)
Production and distribution of industrial foods fortified and enriched with tannins especially hydrolysable tannins, for example, gallotannin	Tannins with antiradical activities and anti‐inflammatory effects can reduce the COVID‐19 disease morbidity and mortality due to their role in redox homeostasis maintenance Gallotannin as hydrolysable tannins showed to inhibit cytokine expression	(Feng & Koh, 2013; Kouhpayeh et al., 2020)
Reduce production and consumption of dairy products because of their relatively high calcium ion content in places with high prevalence of coronavirus Increase production and consumption of all‐bran bakery products because they contain phytic acid as a chelating agent for Fe^2+^, Mn^2+^, Cu^2+^, Mg^2+^, and Zn^2+^ ions Prevent consumption of Fe^2+^, Mn^2+^, Cu^2+^, Mg^2+^, and Zn^2+^ supplements and prevent the production and importing of foods enriched or fortified with these supplements during times and in places of high coronavirus prevalence	The SARS‐CoV structural envelope protein forms calcium ion channels, a novel highly relevant function. Transport of calcium ions through the envelope protein channel stimulates the inflammasome and exacerbates inflammation, worsening the reaction to SARS or SARS‐CoV‐2 infection Overproduction of hydroxyl radicals by Fe^2+^, Mn^2+^, Cu^2+^, Mg^2+^, and Zn^2+^ via the Fenton reaction alters the mitochondria transmembrane potential, inducing the liberation of different apoptogenic factors and subsequent activation of caspase‐3, resulting in disassembly and fragmentation of nuclear chromatin, leading to peripheral blood lymphocytes apoptosis	(Nieto‐Torres et al., 2015; Jimenez‐Guardeno et al., 2014; Nieva et al., 2012; Graham et al., 2013; Triantafilou & Triantafilou, 2014; Elliott & Sutterwala, 2015; Latz et al., 2013; Zhou, Frey, & Yang, 2009) (Del‐Rıo & Velez‐Pardo, 2004; Winter bourn, 1995)

Contrary to the positive role of vitamin D, it can provide increased amounts of extracellular calcium which functions as a potent activator of transient receptor potential channel, melastatin II (TRPM II) (Kouhpayeh et al., 2020). Thus, administration of vitamin D in 2019‐nCoV patients is still controversial.

## 5 VITAMIN C AND STRENGTHENING THE IMMUNE SYSTEM AGAINST β‐CORONAVIRUS

Vitamin C with the use of phagocyte transformation of T lymphocytes and the production of interferon can affect the immune system. In particular, the resistance of chick embryo tracheal organ cultures to infection caused by an avian coronavirus increased by vitamin C. Animal sensitivity to different bacterial and viral infections was modified by vitamin C; for example, in broiler chicks protecting power against an avian coronavirus was improved. Also, the severity of viral respiratory infections or pneumonia in humans is affected by vitamin C levels. In particular, vitamin C increased the resistance of chick embryo tracheal organ cultures to infection caused by an avian coronavirus. Vitamin C is vital to the function of leukocyte white blood cells that help to fight infections and overall immune system health. Vitamin C is also important for iron absorption, and iron insufficiency can enhance vulnerability to infections in general (Hemila, 2003). Therefore, food fortification with vitamin C supplements in times and places of high coronavirus prevalence is strongly recommended (Table 3).

## 6 THE RELATIONSHIP BETWEEN 2019‐NCOV AND NICOTINAMIDE ADENINE DINUCLEOTIDE (NAD^+^), NIACIN (VITAMIN B3), AND L‐TRYPTOPHAN

In the depicted molecular pathology pathway of 2019‐nCoV, almost all procedures lead to or originate from NAD^+^ depletion. NAD^+^ depletion mediated by uncontrolled poly (ADP‐ribose) polymerase (PARP) activity leads to decreased sirtuin 1 (SIRT1) activity indirectly. SIRT1 deacetylates nuclear proteins using NAD^+^ to regulate the expression of genes including tumor suppressors, cytokines, and proto‐oncogenes and ultimately modulate inflammation, cell survival, and apoptosis mechanisms (Kume et al., 2007). NAD and ATP are prerequisite for each other, and consumption of NAD in large amounts decreases ATP levels leading to impairment of all activities and integrity of the cell. In 2019‐nCoV‐mediated acute respiratory distress syndrome (ARDS), aldosterone level is decreased and patients are hypovolemic. It seems that aldosterone synthesis is silenced somewhere in central nervous system (CNS) or adrenal gland. The logical interpretation is serotonin shortage which is an important molecule and has several roles in biology including stimulation of aldosterone secretion (Matsuoka, Ishii, Goto, & Sugimoto, 1985). In 2019‐nCoV patients, the resources of tryptophan—as the raw material for serotonin and NAD synthesis—spend, and in ARDS, course of the disease serotonin is decreased and hypo‐aldosteronism causes hyponatremia and hypovolemia. Fatigue and various degrees of mood disorders are the consequences of NAD, ATP, and serotonin reduction, which could be addressed by concomitant prescription of NAD, Niacin (vitamin B3), and/or its precursor L‐tryptophan with a poly (ADP‐ribose) polymerase (PARP) or poly (ADP‐ribose) glycohydrolase (PARG) inhibitor (Kouhpayeh et al., 2020). Therefore, food fortification with NAD^+^, niacin, and L‐tryptophan in times and places of high coronavirus prevalence is strongly recommended (Table 3). It is possible that administration of NAD alone, along with high activity of PARP and PARG, worsen the clinical manifestation.

## 7 THE RELATIONSHIP BETWEEN 2019‐NCOV AND VITAMIN K

As COVID‐19 may also progress beyond the lungs. Coagulopathy and thromboembolism are prevalent in severe COVID‐19 and relate to decreased survival. Coagulation is an intricate balance between clot promoting and dissolving processes in which vitamin K plays a well‐known role. So that vitamin K status is reduced in patients with severe COVID‐19, and low vitamin K status seems to be associated with accelerated elastin degradation and vitamin K administration improves outcome in patients with COVID‐19 (Dofferhoff et al., 2020). Therefore, Use vitamin k supplements or vitamin K‐rich food sources such as green leafy vegetables, such as kale, spinach, turnip greens, collards, Swiss chard, mustard greens, parsley, romaine, green leaf lettuce, and vegetables such as brussels sprouts, broccoli, cauliflower, and cabbage in food formulations in times and places of high coronavirus prevalence especially for COVID‐19 patients is strongly recommended (Table 3).

## 8 TANNINS (AS A WATER‐SOLUBLE POLYPHENOLIC COMPOUNDS) AND COVID‐19

Tannins were well known as health‐promotor components with antiradical activities and anti‐inflammatory effects, and since the COVID‐19 is considered as an inflammatory disorder, thus tannins as the magic antioxidants can reduce the disease morbidity and mortality due to their role in redox homeostasis maintenance. Gallotannin as hydrolysable tannins showed to inhibit cytokine expression (Feng & Koh, 2013; Kouhpayeh et al., 2020). Herbs and spices such as cloves, tarragon, cumin, thyme, vanilla, and cinnamon; berries such as cranberries, strawberries and blueberries; nuts such as hazelnuts, walnuts, oak, and pecans; legumes such as red‐colored beans; orange‐colored juices such as apple, grape, and berry juices; and chocolate liquor, tea, and wine are tannin‐rich ingredients, and it is strongly recommended to use them in the structure of food formulations in times and places of high coronavirus prevalence (Table 3).

## 9 THE RELATIONSHIP BETWEEN SARS‐COV AND Ca^2+^

As infection is mainly placed on the Golgi apparatus and the endoplasmic reticulum Golgi apparatus intermediate compartment (ERGIC), where it promotes virus production and morphogenesis, the CoV envelope (E) gene encodes a small transmembrane protein extremely produced. Notably, the NF‐κB inflammatory pathway is overstimulated by SARS‐CoV while the E protein is present, and by its PDZ‐binding motif, interacts with the cellular protein syntenin, p38 MAPK activation is triggered (Jimenez‐Guardeno et al., 2014). These signaling cascades result in increased inflammation and immunopathology. One of the most impressive functions presented by the CoV E protein is an ion channel (IC) activity impressing ion transportation, due to forming membrane pentameric protein–lipid pores by the CoV E self‐assembling. Amazingly, lipid head groups are fundamental segments of the pore and regulate ion conductance and selectivity. The SARS‐CoV E protein showed a moderate favorite for cations (Na+, K+) over anions (Cl^‐^) when reconstituted in membranes resembling of the ERGIC/Golgi. In several viral systems, alteration of homeostasis of ions, especially Ca^2+^, is favorable for infection. In this way, an extended variety of viruses encode ion‐conductive proteins similar to E protein, named viro‐porins (Nieva, Madan, & Carrasco, 2012). More than one viro‐porins were encoded by highly pathogenic RNA viruses, for example, coronaviruses, picornaviruses, influenza A virus, and hepatitis C virus, so that in some viruses especially SARS‐CoV resulted in death due to great disruption of the pulmonary epithelium and increasing edema and IL‐1β‐mediated proinflammatory response in the lung parenchyma (Graham, Donaldson, & Baric, 2013; Nieto‐Torres et al., 2015; Nieva et al., 2012). IL‐1β, an efficient proinflammatory cytokine, is essential for obviation infection in severe inflammatory diseases such as asthma, gout, atherosclerosis, and Parkinson's disease. The IL‐1β production in organisms was highly controlled by macromolecular complexes, mainly expressed in macrophages and dendritic cells inherently synthesized in the bronchiolar epithelium, termed inflammasomes (Triantafilou & Triantafilou, 2014). One highly studied inflammasome applicable in the pulmonary tissue is the nucleotide‐binding oligomerization domain (NOD)‐like receptor pyrin domain‐containing protein 3 (NLRP3) inflammasome. This complex is composed of the sensing protein NLRP3, the adapter component apoptosis‐associated speck‐like protein containing a cysteine–aspartate proteases (caspase) activation and recruitment domain, and the catalytically inactive procaspase‐1 (Elliott & Sutterwala, 2015; Latz, Xiao, & Stutz, 2013). The inflammasome components were synthesized under severe danger stimuli, that is, infection state. However, a second signal, that is, ionic imbalances such as Ca^2+^‐imbalance resulted in certain viro‐porins within cells, is consequently needed to induce their assembly, which triggers the NLRP3‐inflammasome that resulted in alternating procaspase‐1 into active caspase‐1, which cleaves inactive pro‐IL‐1β into its mature form, IL‐1β, that is released to the extracellular media to stimulate proinflammation (Elliott & Sutterwala, 2015; Latz et al., 2013; Triantafilou & Triantafilou, 2014). In the mice infected with E protein IC‐proficient SARS‐CoVs, strongly ion conductivity, especially Ca^2+^‐permeable or imbalance in ERGIC/Golgi membranes, was affected consequently stimulating the ERGIC/Golgi inflammasome occurred which led to the overproduction of IL‐1β in the lung airways (Nieto‐Torres et al., 2015). Pharmacological repression of this pathway may develop the basis for combined therapeutics applicable for SARS‐CoV and other viruses (Nieto‐Torres et al., 2015). Therefore, the SARS‐CoV E protein forms channels that are somewhat selective for cations in membranes simulating the ERGIC/Golgi. Within cells, the movement of different cations by the E protein pore should be dictated by their respective gradients. Ca^2+^ possesses the largest asymmetrical distribution between the ER–Golgi lumen (around 100 μM) and the cytoplasm (around 100 nM), which should allow the flow of this cation through the E protein IC channel if it is permeable (Nieto‐Torres et al., 2015). Therefore, in times and places of high coronavirus prevalence, low calcium intake approaches, that is, reduced consumption of dairy products and reduced consumption and production of food products fortified or enriched with Ca^2+^, are strongly recommended (Table 3).

## 10 THE RELATIONSHIP OF SARS‐COV WITH BODY MINERALS, ESPECIALLY Fe^2+^, Mn^2+^, Cu^2+^, Mg^2+^, AND Zn^2+^

The zinc deficiency, at least one‐third of the world population have been affected by zinc deficiency, is responsible for 16% of all deep respiratory infections worldwide can provide a first strong hint on a link of zinc deficiency with the risk of infection and severe progression of COVID‐19 and suggests potential benefits of zinc supplementation (Wessels, Rolles, & Rink, 2020). Administration of Zn supplement has the potential to enhance antiviral immunity, both innate and humoral, and to restore depleted immune cell function or to improve normal immune cell function, in particular in immunocompromised or elderly patients. Zn may also act synergistically when coadministered with the standard antiviral therapy, as was demonstrated in patients with hepatitis C, HIV, and SARS‐CoV‐1. Zn may also protect or stabilize the cell membrane which could contribute to blocking the virus entry into the cell. It was demonstrated that Zn may inhibit viral replication by alteration of the proteolytic processing of replicase polyproteins and RNA‐dependent RNA polymerase (RdRp) in rhinoviruses, HCV, and influenza virus, and diminish the RNA‐synthesizing activity of nidoviruses, for which SARS‐CoV‐2 belongs. Therefore, it may be hypothesized that Zn supplementation may be of potential benefit for prophylaxis and treatment of COVID‐19, especially for those who have a Zn deficiency (Kumar, Kubota, Chernov, & Kasuya, 2020).

On the other hand, the Fenton reaction catalyzed by iron, manganese, copper, and zinc causes apoptosis in peripheral blood lymphocytes with the production of hydrogen peroxide (H_2_O_2_) and the hydroxyl radical (OH). Both oxidizing compounds (i.e., H_2_O_2_ and OH) cause depolarization of mitochondria, following caspase‐3 activation, and chromatin condensation/fragmentation, representative of apoptosis. Distinctly, apoptosis and the highest necrosis were induced by 250 µM zinc and 1,000 µM metal ion, respectively. Similar morphologic changes and percentages of apoptosis were observed when cells were exposed to 50–1,000 µM iron, manganese, and copper ions. Apoptosis and necrosis were induced by the redox‐active metals, depending on metal concentration. The same morphologic changes and apoptosis percentage were revealed while cells were exposed to 50–1000 µM Fe^2+^, Mn^2+^, Cu^2+^, Mg^2+^, and Zn^2+^ (Del‐Rio & Velez‐Pardo, 2004). Therefore, in times and places of high coronavirus prevalence, especially for those who do not have a deficiency of these minerals, low body mineral intake approaches, that is, reduced consumption and production of food products fortified or enriched with these minerals and increased consumption of bakery goods containing whole bran to reduce dietary mineral absorption, are strongly recommended (Table 3).

(1) $${\text{Fe}}^{2+}+H_{2}O_{2}\to {\text{Fe}}^{3+}+\left[H_{2}O_{2}^{‐}\right]\to^{∙}\text{OH}+{\text{OH}}^{‐}$$

(2) $${\text{L-Fe}}^{2+}+H_{2}O_{2}\to \text{L-Fe}{\left(H_{2}O_{2}\right)}^{2+}$$

(3) $$\text{L-Fe}{\left(H_{2}O_{2}\right)}^{2+}\to {\text{L-Fe}}^{3+}+\text{OH}+{\text{OH}}^{‐}$$

(4) $$\ldots \ldots \ldots \to {\text{L-Fe}}^{4+}+{\text{2OH}}^{‐}$$

(5) $$+R\to {\text{L-Fe}}^{3+}+{\text{OH}}^{‐}{+}^{∙}\text{ROH}$$

(Fenton reaction for ferrous ions in the body)

## 11 CONCLUSIONS

Appropriate approaches to food production technology should be considered including avoiding using any abnormal animal meat that could be a potential source of β‐coronavirus transmission; following good food safety practices in food production lines; avoidance of underheating in the processing of swine and other meat products; uncertainty about the safety of frozen or refrigerated meat products; and providing unfavorable environmental conditions for coronavirus survival (minimum heat treatment, e.g., LTLT and more for liquid food products, pH ≤ 3, minimum storage relative humidity); production of industrial foods fortified and enriched with vitamin D, C, B3, K, amino acid L‐tryptophan, nicotinamide adenine dinucleotide (NAD^+^), and tannins especially hydrolysable tannins, for example, gallotannin; and finally, preventing the production of industrial foods enriched or fortified with mineral supplements that participate in the Fenton reaction in the human body.

## CONFLICT OF INTEREST

The authors declare that they do not have any conflict of interest, and the study did not involve any human or animal testing.
